# Novel transcripts discovered by mining genomic DNA from defined regions of bovine chromosome 6

**DOI:** 10.1186/1471-2164-10-186

**Published:** 2009-04-24

**Authors:** Rosemarie Weikard, Tom Goldammer, Annett Eberlein, Christa Kuehn

**Affiliations:** 1 Forschungsinstitut für die Biologie landwirtschaftlicher Nutztiere (FBN), 19196, Dummerstorf, Germany

## Abstract

**Background:**

Linkage analyses strongly suggest a number of QTL for production, health and conformation traits in the middle part of bovine chromosome 6 (BTA6). The identification of the molecular background underlying the genetic variation at the QTL and subsequent functional studies require a well-annotated gene sequence map of the critical QTL intervals. To complete the sequence map of the defined subchromosomal regions on BTA6 poorly covered with comparative gene information, we focused on targeted isolation of transcribed sequences from bovine bacterial artificial chromosome (BAC) clones mapped to the QTL intervals.

**Results:**

Using the method of exon trapping, 92 unique exon trapping sequences (ETS) were discovered in a chromosomal region of poor gene coverage. Sequence identity to the current NCBI sequence assembly for BTA6 was detected for 91% of unique ETS. Comparative sequence similarity search revealed that 11% of the isolated ETS displayed high similarity to genomic sequences located on the syntenic chromosomes of the human and mouse reference genome assemblies. Nearly a third of the ETS identified similar equivalent sequences in genomic sequence scaffolds from the alternative Celera-based sequence assembly of the human genome. Screening gene, EST, and protein databases detected 17% of ETS with identity to known transcribed sequences. Expression analysis of a subset of the ETS showed that most ETS (84%) displayed a distinctive expression pattern in a multi-tissue panel of a lactating cow verifying their existence in the bovine transcriptome.

**Conclusion:**

The results of our study demonstrate that the exon trapping method based on region-specific BAC clones is very useful for targeted screening for novel transcripts located within a defined chromosomal region being deficiently endowed with annotated gene information. The majority of identified ETS represents unknown noncoding sequences in intergenic regions on BTA6 displaying a distinctive tissue-specific expression profile. However, their definite regulatory function has to be analyzed in further studies. The novel transcripts will add new sequence information to annotate a complete bovine genome sequence assembly, contribute to establish a detailed transcription map for targeted BTA6 regions and will also be helpful to dissect of the molecular and regulatory background of the QTL detected on BTA6.

## Background

Independent linkage analyses consistently indicate several QTL for production, health and conformation traits on bovine chromosome 6 (BTA6) [1-3]. Prerequisite to elucidate the molecular background causing the genetic variation at the QTL is a detailed, well-annotated gene and transcript sequence map of the chromosomal regions covering the QTL intervals. Particularly, functional genomic studies in chromosomal regions containing QTL rely on transcript sequences to create expression microarrays and perform QTL expression studies. Through the efforts of the bovine genome projects the currently identified genes are primarily derived from large-scale sequencing efforts in combination with sequence comparison approaches to available gene sequences from sequence-ready genomes as mice and human. Comparative mapping of genes from the syntenic human chromosome 4 (HSA4) on BTA6 by high-resolution radiation hybrid mapping was performed [4-10] helping to identify genes with orthologous counterparts on BTA6. However, it is not clear, which proportions of species-specific genes could have been missed by such efforts. Missing genes may be due to the fact that there are a number of genes in individual mammalian genomes, which are expressed at a very low level or only in a time-limited or tissue-specific manner at defined developmental stages, and which are difficult to be isolated by conventional cloning techniques.

Moreover, there is increasing indication for the existence of lineage- or species-specific transcripts from numerous studies (e.g., [11]). Recent studies [12-15] provided conclusive evidence for the abundance of lineage-, tissue-, development-, or spatial-temporal-specific transcripts encoded in the cattle genome. For instance, Kumar and colleagues [12] discovered a number of novel transcripts predominantly expressed in the cattle placenta, which not had been found previously in primates or rodents. These results indicate towards the molecular basis of the relative anatomical variation in placental architecture of ruminants and suggest that loss and gain of genes and transcript variants are important mechanisms of genome evolution in mammals. Especially in ruminants, there is already unequivocal evidence for the occurrence of highly divergent genes encoding proteins having adaptive functions in the biology and metabolism of reproduction (e.g., IFN tau, [16]).

Given the unique adaptations for the ruminant reproductive system, it is conceivable that also other highly divergent processes associated with rumination and dramatic adaptive changes in ruminant metabolism, for instance, at the onset of lactation and continuous maintenance of a high milk production performance [17-21] could be caused by the action of individual species-specific genes or transcripts. This would provide important rationale for the identification of genes or transcripts being located in QTL intervals associated with milk production traits.

To complete the bovine gene catalogue on the targeted BTA6 region carrying the QTL, alternative methods will be required to identify novel transcripts and genes independently of their individual tissue-specific or spatial-temporal expression pattern. In our study, we focused on targeted isolation of transcribed sequences from defined subchromosomal regions on BTA6 poorly covered with gene information. Therefore, we exploited bovine bacterial artificial chromosome (BAC) clones mapped to the QTL interval using exon trapping, a technique that has been developed to identify genes in cloned eukaryotic DNA (e.g., [22-24]). This technique enables the experimental identification of potential exons in a fragment of eukaryotic DNA of unknown gene content or intron-exon structure based on the presence of functional splice sites; thus making the exon trapping assay independent of the expression pattern of the transcripts to be identified. This feature represents the main advantage of the exon trapping method compared to other methods of gene identification (cDNA selection [25-27] or cDNA hybridization approaches [28-30]).

Identified putative transcript sequences isolated from BTA6-specific BAC clones *in vitro *using the exon trapping technique were subjected to a comprehensive sequence comparison analysis by alignment to sequence assemblies of the bovine, human and mouse genomes. The genuine presence of the identified exon trapping sequences (ETS) in the bovine transcriptome was verified by multi-tissue expression analysis using RT-PCR.

## Results

**Figure 1:**
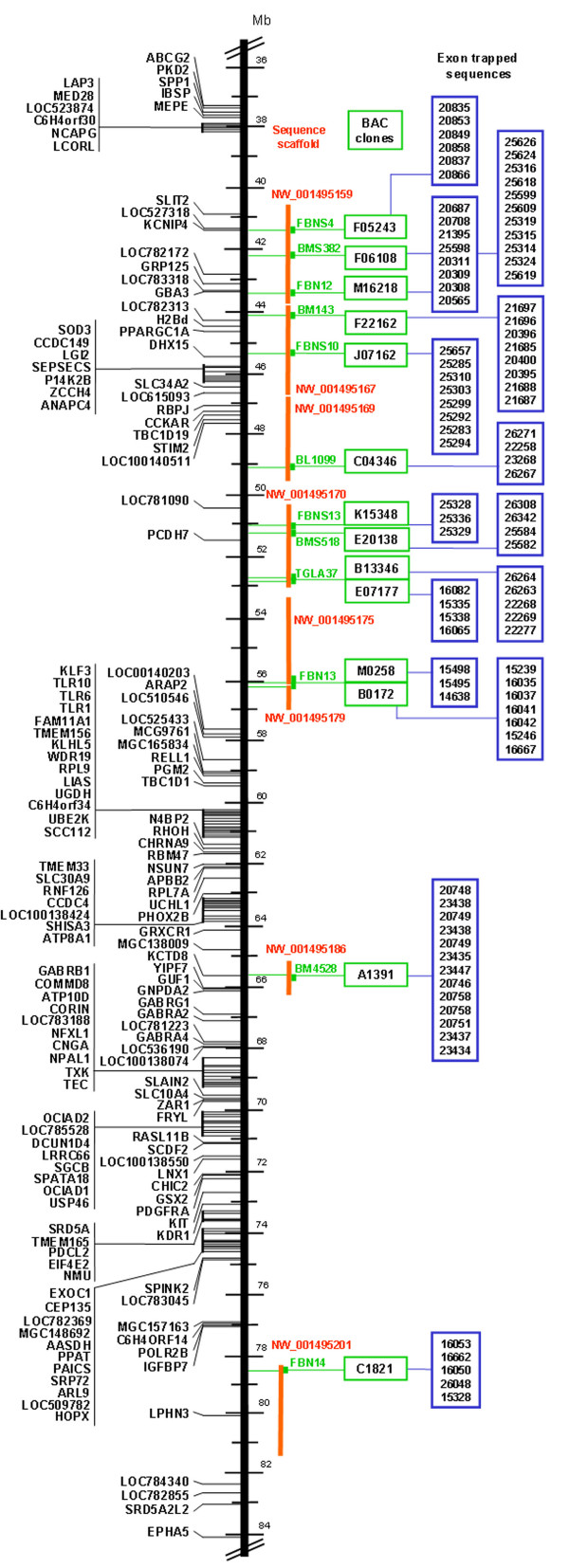
**Chromosomal localization of exon trapping sequences on bovine chromosome 6 (BTA6)**. Left: Protein-coding genes assigned to BTA6 (bovine genome sequence assembly Btau4.0). Right: Markers used for BAC library screening (green). BAC clones (green-framed boxes). Bovine genomic scaffolds (red). Exon trapping sequences (blue-framed boxes).

As an initial step towards systematic transcript analysis in the targeted BTA6 region we performed exon trapping on fourteen selected chromosome region-specific BAC clones that mainly mark the segment between BTA6q16 and BTA6q24 and correspond to defined subchromosomal regions between 41.4 and 78.5 Mb on the sequence assembly for BTA6 (NCBI, Btau4.0) as shown in Figure 1. For each BAC clone two exon trapping libraries were generated based on both, completely *BamHI/BglII *and partially *Sau3AI *restriction enzyme digested BAC DNA. To eliminate the majority of sequences due to vector self splicing or cryptic splicing events, we employed PCR screening of clones from the exon trapping libraries followed by size selection of PCR products prior to sequencing. From each library 30 randomly selected clones were subjected to PCR with insert-flanking vector-derived primers and a medium of 14 clones from each exon trapping library were selected for sequencing. Thus, a total of 396 putative exon trapping sequences (ETS) with a size varying from 35 to 349 bp were identified.

Sequence comparison identified 92 sequences as being derived from the trapping vector caused by vector self-splicing and from the BAC vector sequence or due to cloning artefacts, which indicated 23% false positive splicing events. Of the remaining 304 putative ETS, 92 (30%) were unique sequences. The number of unique sequences detected per BAC clone varied between three and fifteen. There was a relatively high percentage (70%) of ETS observed redundantly. Some sequences were found with a frequency up to fourteen-fold. This redundancy is due to the fact that for each BAC clone two separate exon trapping libraries based on two different restriction enzyme digestion of BAC DNA were constructed. However, the set of ETS detected in the two single libraries constructed per BAC clone was not completely identical. Furthermore, it has also to be considered that the exon trapping technique itself includes several steps based on PCR amplification, a feature that intrinsically should be associated with the abundance of multiple copies of specific DNA targets.

The size of unique putative ETS ranged from 35 to 317 bp with a medium of about 125 bp. Locus-specific primers were derived for unique putative ETS with a size exceeding 60 bp (see Additional file 1). These primers were applied to remapping of ETS to their parent BAC clones by PCR. The chromosomal localization of exons on BTA6 could be inferred from physical mapping of the BACs carrying the trapped sequences by FISH or by *in silico *sequence similarity mapping of the corresponding BAC end sequences on the genome sequence assembly of BTA6 in combination with our high-resolution RH map [9].

**Figure 2:**
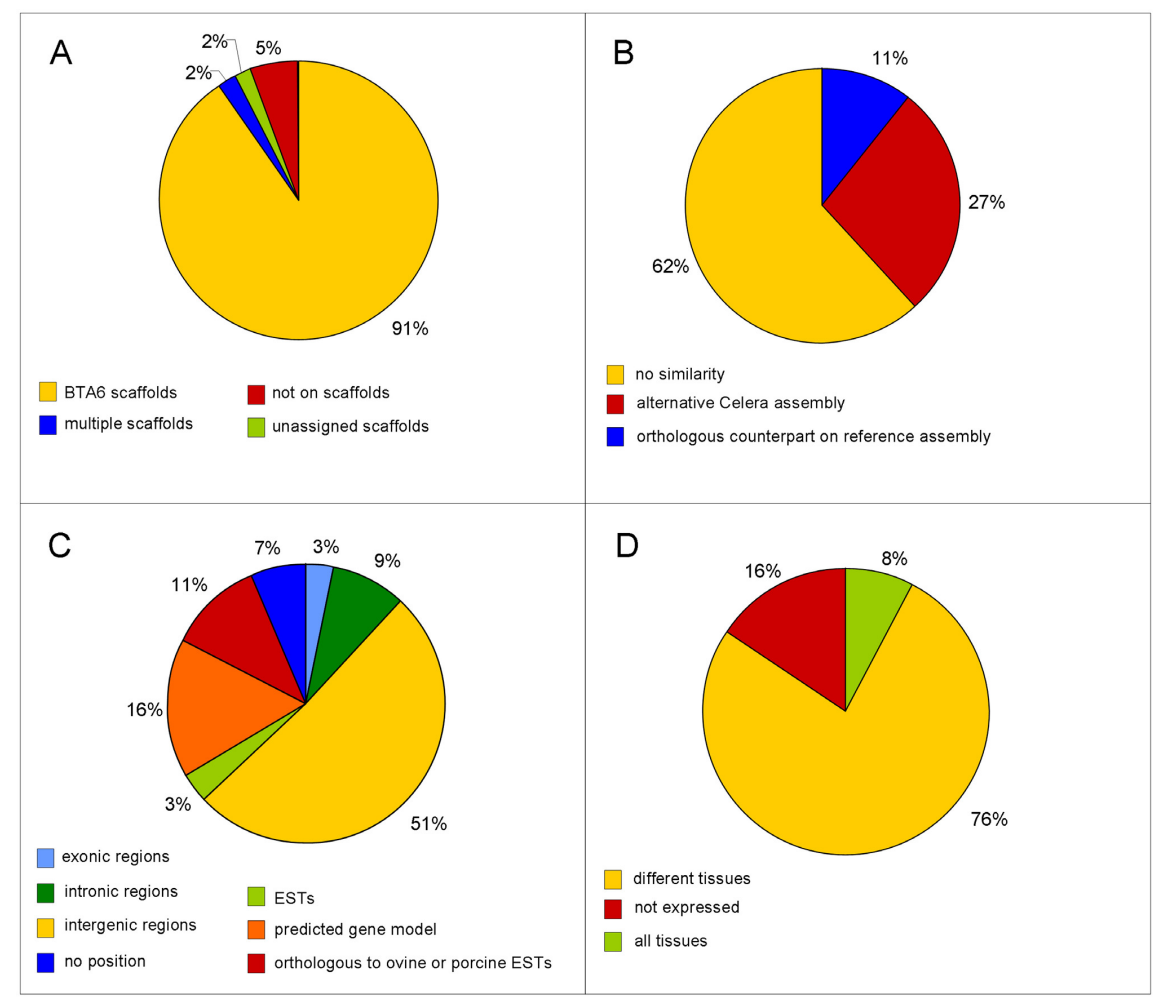
**Analysis of transcripts isolated from gene-poor regions on bovine chromosome 6 (BTA6) by exon trapping**. A: Assignment of exon trapping sequences (ETS) to the bovine genome assembly Btau4.0. B: Sequence similarity of ETS to genome sequence assemblies from human and mice. C: Distribution of ETS to the annotated bovine genome. D: Expression of ETS analyzed in a multi-tissue panel of a lactating cow.

All 92 unique ETS were further analyzed by screening the bovine genome sequence assembly (NCBI, Btau4.0) for similarity to known sequences, genes or transcripts. The results are summarized in Figure 2A and presented in detail in Additional file 2. Sequence identity to sequence scaffolds on BTA6 was detected for the majority of unique ETS (91%). Two ETS were identified on sequence scaffolds not yet assigned. A further two sequences contained repetitive sequence motifs and matched to more than one scaffold located on different chromosomes. We found that five ETS (5%) could not be assigned to the bovine genome sequence resources by *in silico *sequence similarity search.

As illustrated in Figure 2B and presented in Additional file 2 in more detail, for a number of ETS (28%) sequence similarity was found to genomic sequence scaffolds from alternative sequence assembly of the human genome (based on Celera assembly, for instance ETS_25316, ETS_25598 etc.). Furthermore, a few ETS (11%) revealed similarity to genomic sequences located on the syntenic chromosomes HSA4 and MMU5 of the human and mouse reference genome assemblies. The remaining ETS did not detect comparative genomic counterparts in human and mouse.

According to Additional file 2 and Figure 2C, we found that only a small number of ETS showed identity to known bovine cDNA sequences or expressed sequence tagged sites (ESTs). On BAC clone BBI_750F0243 carrying ETS_20835, ETS_20849 and ETS_20866, we identified four exons of the bovine KCNIP4 gene (NM_001076935), which has an orthologous counterpart on the syntenic HSA4 region. The ETS_20866 sequence was not yet identified on scaffolds of the current bovine sequence assembly, although the mRNA of the bovine KCNIP4 gene revealed 100% identity to this sequence, which is also supported by sequence identity of ETS_20866 to several bovine ESTs. Strong sequence similarity of ETS_20866 was also identified to the adjacent exons 11 and 12 of the orthologous human gene. This result indicates that the bovine KCNIP4 gene is not completely annotated in the current bovine sequence assembly because at least two exons are missed in the predicted gene model. Besides to human, high comparative sequence similarity of these ETS was also observed to exons of the KCNIP4 gene from other species (rat, mouse, chicken and horse).

Sequence identity to known bovine ESTs was detected for ETS_25609 on BAC BBI_750F06108, ETS_15246 on BAC BBI_750B0172, and ETS_25294 on BAC BBI_750J07162. The ESTs were all assigned to sequence scaffolds on BTA6 (NW_001495159, NW_001495179, and NW_001495167, respectively), where the corresponding parental BAC clones were assigned to. In addition, ten ETS captured from BAC clone BBI_75006108 revealed sequence similarity to two ovine mRNAs and one ETS from BAC clone BBI_750B0172 showed similarity to a porcine transcript.

To analyze their potential protein-coding capacity, the unique ETS were screened for existing open reading frames (ORFs) using the program ORF Finder at NCBI. For most ETS, the program could predict ORFs. However, no ORF spanned the sequence length of the analyzed ETS completely. Protein coding sequences predicted by the program were subjected to sequence alignment with TBLASTN in the nucleotide collection and the ESTs databases at NCBI. Again, only the sequences ETS_20835, ETS_20849 and ETS_20866 showed high similarity matches pointing to the KCNIP4 gene from several species.

Including the *ab initio *map at the NCBI *Bos taurus *MapViewer [31] into our analysis we identified fifteen ETS with sequence similarity to bovine gene models predicted by the gnomon gene prediction program. One of them (ETS_16667) pointed to a hypothetical locus (LOC100140872) predicted to be a pseudogene similar to the PHYHIPL gene (see Additional file 2, Figure 2C).

The results obtained by the different sequence similarity analyses suggested that the majority of the isolated transcripts in our study presumably represent bovine noncoding transcripts located in intergenic regions. By screening the comprehensive mammalian noncoding RNA database (RNAdb 2.0) we could not identify similarity to known noncoding RNAs in the available data sets [32].

To elucidate, if the identified ETS were really expressed in the bovine transcriptome, multi-tissue expression analysis by RT-PCR using locus-specific primers on total RNA from nine different bovine reference tissues (liver, small intestine, kidney, thyroid gland, mammary gland, pituitary gland, skeletal muscle, subcutaneous and intestinal fat) was performed. For each BAC clone we exemplarily analyzed at least three ETS, if available and specific primers could be designed.

**Figure 3:**
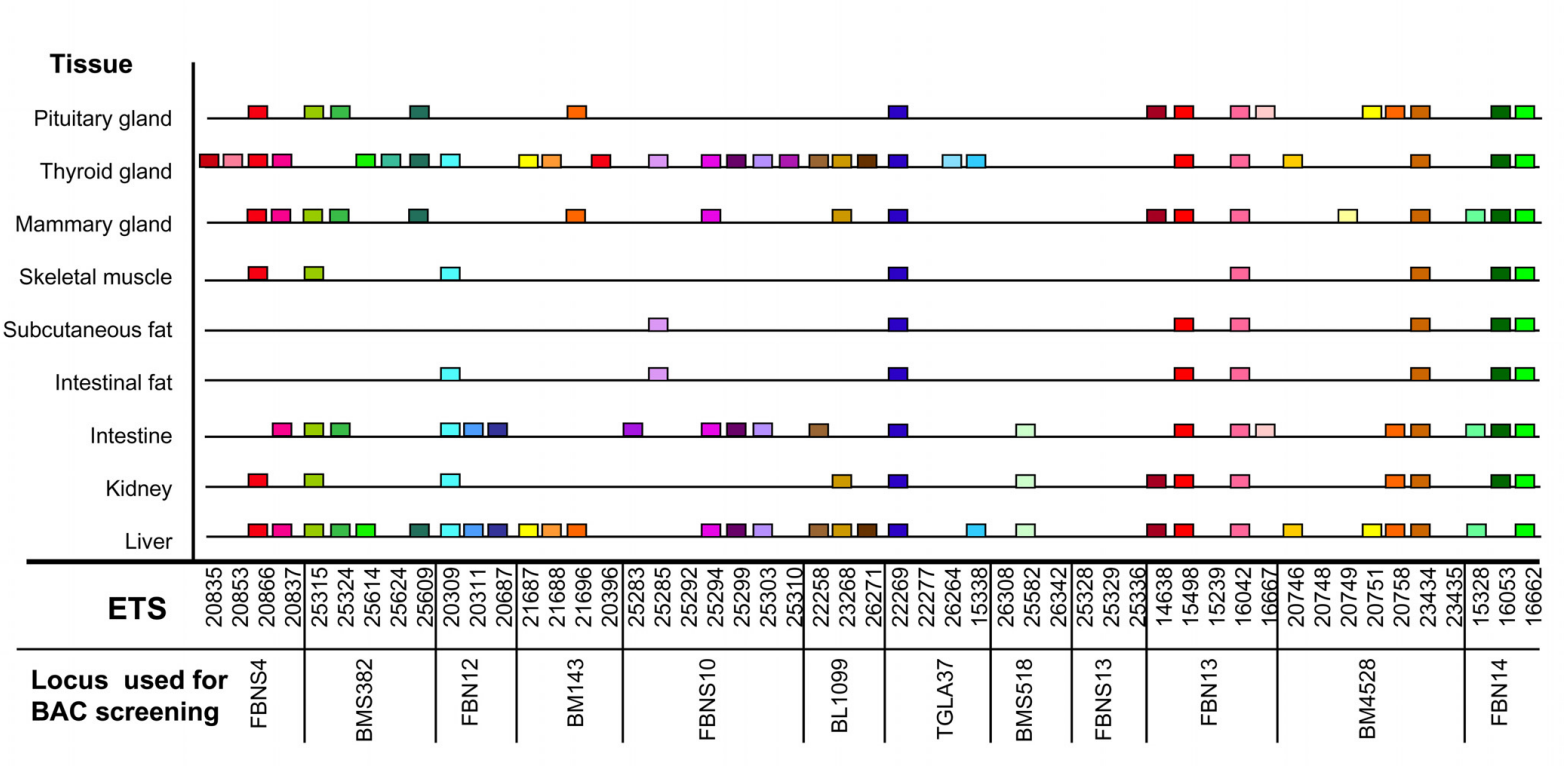
**Tissue-specific expression pattern of sequences isolated by exon trapping on BAC clones specific to bovine chromosome 6 (BTA6)**. Y-axis: Tissues of a lactating cow. X-axis: Exon trapped sequences (ETS) ordered to the marker loci used for BAC library screening. Expression in a specific tissue was analyzed by RT-PCR and is displayed by a coloured box specific for each ETS.

The results are summarized in Figure 2D and are displayed in detail in Figure 3. They show that 43 ETS out of 51 analyzed (84%) were expressed at least in one tissue isolated from a lactating cow. Specifically, 39 ETS displayed a different expression pattern in the tissues analyzed, and four revealed expression in all tissues examined.

**Figure 4:**
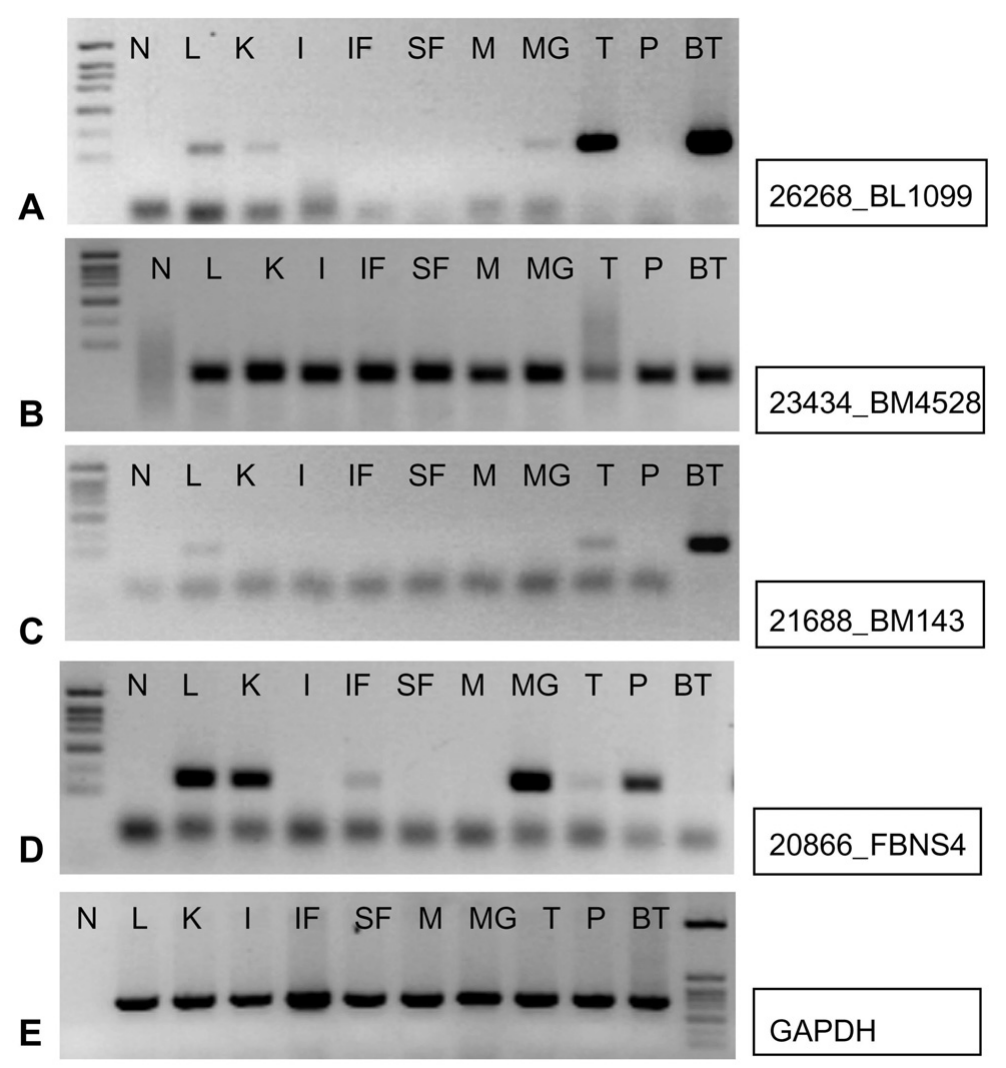
**Expression of selected exon trapped sequences (ETS) on a bovine multi-tissue panel analyzed by RT-PCR**. A, C, D: examples for different tissue-specific expression, B: example for ubiquitous tissue expression. E: expression of GAPDH as control house-keeping gene. Sequences analyzed are given right to each electrophoresis profile. L: liver, K: kidney, I: small intestine, IF: intestinal fat, SF: subcutaneous fat, M: skeletal muscle, MG: mammary gland, T: thyroid gland, P: pituitary gland, BT: bovine genomic DNA, N: no template control.

In Figure 4(A–D), examples of different expression patterns of ETS ranging from expression in only a few tissues (A, C, D) to ubiquitous expression (B) observed in the experiment are shown. Figure 4E displays expression of the house-keeping gene GAPDH in the tissues analyzed in our study. Generally, ETS revealed amplification of the expected amplicon size in bovine genomic DNA (see Figure 4A–C, lane BT). However, in the case of ETS_20866 amplification in bovine genomic DNA failed (Figure 4D, lane BT), which is due to the fact that this ETS consists of two adjacent exons. This was supported by sequence comparison to the human KCNIP4 gene sequence revealing that the primers of ETS_20866 span an intronic region on genome level. There were a further 6 unique ETS captured by exon trapping (ETS_21696, ETS_25582, ETS_16037, ETS_15328, ETS_25303, ETS_26263), which also seem to contain at least two adjacent transcripts and are interrupted by intergenic DNA sequence on genomic sequence level. This result is supported by both alignment of the identified sequences to sequence scaffolds and experimentally, by amplification of the corresponding longer amplicons on bovine genomic DNA.

## Discussion

The main goal of the Bovine Genome Project, the identification of the whole bovine genome sequence, is almost achieved [33], but still there are several regions in the bovine genome not sufficiently annotated and characterized. Genome assemblies also rely on the existence of transcript sequences to merge contigs together, verify the assembly of whole genome shotgun reads, and annotate genes. Further analysis of QTL regions of interest may include physical and transcription mapping, identification of positional and functional candidate genes and isolation of the corresponding full-length cDNA as well as association studies on the selected genes.

This study represents a further step in the ongoing molecular and genetic analysis of complex traits and annotation of genes and transcripts localized in the region on BTA6 containing QTL for milk and meat production, health and conformation traits.

As an initial step towards systematic analysis of transcripts and genes in this region, we carried out exon trapping using selected bovine BAC clones previously mapped to QTL intervals on BTA6. Mining a genomic interval comprising about 1 Mb for transcribed sequences using this technique, we identified a total of 92 unique exon trapping sequences. Genome similarity searches revealed sequence identity matches to sequence scaffolds on BTA6 for most unique ETS (91%). With two ETS, which were identified on sequence scaffolds not yet assigned to the current NCBI sequence assembly of the bovine genome, gaps could be closed on the genome sequence level. There were about 2% of ETS, which could not be unambiguously assigned to the bovine genome sequence, because of matches to multiple chromosomes due to repetitive sequence motifs. We found only 5% of ETS without any hits to known sequences contained in the archive of bovine sequence databases. These ETS can provide new additional sequence information to complete the current genome sequence assembly. This result further indicates that targeted deep sequencing within the corresponding genomic regions would be required to improve the accuracy of the BTA6 sequence assembly.

Comparative sequence similarity search to human and mouse genome sequences revealed that 11% of the isolated ETS displayed high similarity to genomic sequences located on the syntenic chromosomes HSA4 and MMU5 of the human and mouse reference genome assemblies pointing to highly conserved genome regions in these species. Almost a third of the ETS identified similar equivalent sequences in genomic sequence scaffolds from the alternative Celera-based sequence assembly of the human genome. The residual 62% of ETS without comparative genomic sequence counterparts in human and mouse refer to presumably species-specific genomic regions in the bovine genome.

Screening the gene, ESTs and protein databases at NCBI detected only a few known transcribed sequences revealing identity to the ETS isolated by exon trapping in our study (17%, Figure 2C).

Whereas 6% of all ETS identified known bovine transcripts, a further 16% of ETS pinpointed to bovine gene models predicted *ab initio*.

For the evaluation of the relatively low proportion of ETS identifying known transcripts, we have to consider, that the BAC clones subjected to exon trapping in our study had been selected from regions on BTA6 poorly covered with protein-coding genes, which could still be noted on the current sequence assembly Btau4.0 (Figure 1). Eleven BACs were assigned to gene desert regions [9], which have been found conserved in mammals and birds [34,35] and were thought to be transcriptionally silent. Hence, we could not expect *a priori *that many sequences would have been annotated as known genes. It should also to be considered that even the current annotation of the bovine genome is still limited and consequently, the set of functional elements is not completely identified to date. Additionally, transcripts from the most lowly expressed genes, or genes specifically expressed in important but relatively minor cell types may very likely be under-represented in the ESTs database predominantly established by large scale ESTs projects.

At the beginning of our experiments it was known that only one BAC clone (BBI_750F0243) was assigned near a human gene (KCNIP4) on the syntenic region on HSA4 by *in silico *comparative mapping. Thus, this BAC clone could serve as a proof for the efficacy of the exon trapping procedure. Indeed, we identified ETS in this BAC clone pinpointing to four exons of the bovine KCNIP4 gene, which underlined and validated the usefulness of the exon trapping method for targeted mining of transcribed sequences in defined chromosomal regions based on genomic DNA from BAC clones. In addition, by identifying two additional exons of the bovine KCNIP4 gene, which are not present in the current bovine genome assembly Btau4.0, it was exemplarily demonstrated that this experimental approach is a useful complement for the annotation of the bovine genome sequence.

Because there was no identity detected to known genes and ESTs by *in silico *sequence comparison for the majority of ETS identified in our study, these sequences are assumed to be novel and could be predicted to originate from unknown bovine transcripts. Expression analysis was performed to validate this hypothesis. Examination of a subset of the trapped putative transcripts showed exemplarily that in a lactating cow numerous ETS displayed a divergent, tissue-specific expression pattern (Figure 2D, Figure 3). Most expression signals were observed in liver, thyroid gland, small intestine, kidney, and pituitary gland. Tissue-dependent expression pattern of the ETS may indicate to potentially specific functions in the corresponding tissues of the lactating cow. Some of the ETS were found to be expressed in all tissues examined, indicating a ubiquitous expression pattern and suggesting them being probably part of housekeeping genes or conserved structural genes, if similarity to repetitive sequences could be excluded. As shown in Figure 3, 16% of the analyzed ETS did not display expression signals in the multi-tissue panel. This could likely be due to the fact, that these transcripts were not expressed in the tissues contained in the panel of a lactating cow analyzed here. In this context it should be mentioned that the advantage of the exon trapping approach is that the method is independent of spatio-temporal expression patterns due to the identification of transcripts based only on intrinsic characteristics of the genuine genomic sequence. But non-expressed ETS, possibly, could also represent false-positive sequences isolated from regions of the genomic DNA due to existing sequence similarities to splice site consensus sequences (e.g., splice donor/acceptor, branch point region), which the exon trapping technique is based on.

From the results of the expression analysis it could be inferred that the ETS revealing expression in the bovine multi-tissue panel may represent *bona fide *transcribed sequences. However, the ETS have to be characterized in further studies with regard to their functional significance. Based on the presented data, we can not exclude that a part of the identified ETS may be attributed to the class of pseudogenes or to non-functional RNA. Pseudogene transcription has been observed in small-scale gene-centred studies and genome-scale unbiased mapping of transcriptionally active regions in the human and mouse genomes. Surveys of Gerstein and Zheng [36,37] have revealed that for example, 5–20% of human pseudogenes can be transcriptionally active. However, considering the relatively high percentage of ETS (84%), for which expression has been demonstrated in our study, it could be assumed that a number of them might attain to another category of transcribed sequence elements as for example noncoding RNA.

Continued submission of ESTs and other sequence information in a variety of species points to the existence of transcripts that do not map to currently annotated genes [12,38-40]. These transcripts may possibly correspond to novel protein coding genes, genes encoding small unknown peptides, pseudogenes or noncoding RNA. Evidence of transcription had increasingly been found in unannotated intergenic genome regions of the human genome, which were thought to be transcriptionally silent (e.g., [41-46]). The ENCODE consortium reported that a vast amount of DNA, not annotated as known genes, is transcribed into RNA. While the majority of the genome appears to be transcribed at the level of primary transcripts, only about the half of the processed transcripts is mapped as currently annotated genes [36,42,46]. Particularly, a high number of new transcriptionally active regions (more than 50%) were detected in non-annotated intergenic regions. These studies indicated that genomic regions previously considered as "junk" encode for multiple polyadenylated and non-polyadenylated transcripts of unknown function. According to Gerstein et al. [36] the ENCODE project provided evidence that there is much activity between annotated genes and intergenic space in the human genome contributed by transcribed non-protein-coding RNAs and transcribed pseudogenes. The authors highlighted that a number of these transcribed pseudogenes and noncoding RNA genes are located even within introns of protein-coding genes and assumed that these components may possibly influence the expression of their host genes. It is also possible that these transcripts themselves do not have a direct function, but rather are important for a particular process (e.g., chromatin accessibility for transcription factor binding). Continuously, numerous noncoding RNA sequences are recognized in the transcriptomes of different eukaryotes as having important regulatory functions in controlling various levels of gene expression in physiological and developmental processes and diseases of complex organisms (e.g., [44,47-49]). The detailed investigation of the functional relevance of the numerous unknown transcripts was postulated as a prospective task in the post ENCODE era.

The findings of our study provide experimental support for transcripts lacking ESTs or other cDNA evidence in the targeted regions of BTA6. The majority of unknown ETS presumably identified novel noncoding transcripts located in intergenic regions of the chromosome. However, prospective studies should be performed to further characterize the transcripts with regard to their putative functional significance. In this respect it has to be proven, if these transcripts belong to non-functional RNA or if they have any specific regulatory function in the bovine genome. Currently, there is scare information on the function of bovine noncoding RNA genes compared to the state in mouse and human. The prevalence of bovine noncoding RNAs, their regulatory impact on gene expression and their physiological effects are not yet examined in detail.

While the mammalian genomes contain nearly similar repertoires of protein-coding gene sequences comprising only a fraction of about 1.5% of the whole genome, the majority of the mammalian genome is obviously transcribed. Our results support the increasingly accepted concept suggesting that the physiological complexity and the unique phenotypes of species-specific or individual genomes might evolve from combinatorial features contributed by the entire genome sequence including previously neglected genome regions [33,42,45,46,50]. Consequently, variation in noncoding sequences might be important effectors of phenotypic variation in complex traits and diseases (see reviews [51,52]) in livestock.

The results of our study on BTA6 demonstrate that the exon trapping method based on region-specific BAC clones is applicable to targeted screening for novel transcripts located within a defined chromosomal region sparsely covered with annotated genes. The novel transcript sequences obtained will contribute to establish a detailed transcription map for targeted specific subchromosomal BTA6 regions. Our results show that the computational prediction and identification of genes and transcripts and manual inspection solely are not sufficient to annotate the final bovine genome in the absence of experimentally derived data. Experiences from genome studies in other species revealed that genome annotation is never complete or final (e.g., [39,40,42,45,53,54]). Therefore, correcting and refining the genome annotation is a reiterative task, which is continuously being done and depends on experimental data for final validation, especially for the identification of rare transcripts and alternative splice variants. Compared to high-throughput sequencing technologies like transcriptome sequencing initiated currently, the method of exon trapping has some advantages. Detection of transcripts by high-throughput sequencing requires knowledge about the temporal-spatial expression pattern of the targeted group of unknown transcripts. Frequently, the time point of expression is difficult to predict, e.g., for transcripts of high relevance for developmental regulation. Furthermore, high-throughput technologies have their limits regarding detection of rare transcripts. Therefore, a targeted approach independent of amount, time and locus of expression using a method like exon trapping will complement high throughput technologies for the analysis of defined chromosomal intervals, for example to trace transcripts in fine-mapped QTL regions.

## Conclusion

In this context, the identification of novel transcripts for BTA6 can contribute to improve the accuracy of gene annotation of the bovine genome and provide new, additional sequence information to help to complete the current genome sequence assembly. Hence, the data obtained in this study was submitted to the database of the annotation community of the bovine genome [55] for implementation in the current international efforts of annotation of the bovine genome. The major objective of these activities is to generate a set of manually annotated bovine genes, connect them to biological function and incorporate them into the Bovine Official Gene Set, which will be made available at NCBI.

## Methods

### BAC library screening and identification

DNA superpools of bovine BAC libraries [BBI_B750, available at ImaGenes, the former German Resource Center for Genome Research (RZPD) Berlin, [56] were screened by PCR as described previously [9]. Primers of 12 loci including nine microsatellite markers (FBN12, BM143, BMS382, BL1099, TGLA37, BMS518, FBN13, FBN14, BM4528) and three targeted sequence tagged sites (FBNS4, FBNS10, FBNS13) isolated from a microdissection library specific for the BTA6 region [57] were used for the identification of specific BAC clones. These loci, which were previously mapped on BTA6 by radiation hybrid (RH) mapping [8], were selected according to their position in the QTL interval and the poor gene coverage within the chromosomal region they were mapped to. Information concerning primer sequences and references of these loci is given by Weikard et al. [8]. The specificity of the identified BAC clones was determined by direct BAC sequencing with primers used for BAC library screening and/or Fluorescence *in situ *hybridization (FISH) [58]. BAC DNA extractions were performed using the Qiagen Large-Construct Kit (Qiagen, Hilden, Germany) according to the manufacturer's instructions.

The genome coverage of the of BAC clones was estimated from *in silico *similarity search of BAC end sequences [9] on the bovine genome sequence assembly of BTA6. Calculating insert sizes of BAC clones by *in silico *mapping on the basis of the recent sequence assembly (NCBI, Btau4.0) revealed experimentally unachievable insert sizes for two clones (BBI_750K15348: of 865 Kb and BBI_750M0258: 198 Kb, see Additional file 2), which could possibly be due to errors in the sequence assembly. Therefore, medium inserts sizes were applied for these two clones in our estimation of genome coverage.

### Exon trapping on BAC clones

Exon trapping relies on the conservation of cis-acting sequences at intron-exon boundaries required for splicing in all eukaryotic species. By subcloning a genomic fragment into the intron of an expression vector, internal exons encoded in the genomic fragment will be spliced into the transcript encoded on the expression vector. Successful capture of exons by internal exon trapping relies on the prerequisite, that the cloned genomic DNA fragment focused on, contains at least one exon flanked by intronic sequences [22,23,59]. Reverse transcriptase polymerase chain reaction (RT-PCR) using primers specific for the transcript on the expression vector will provide a product for analysis by electrophoresis and sequencing.

Essentially, exon trapping was performed using the exon trapping system formerly provided by Gibco/Life Technologies (now Invitrogen) according to the manufacturer's instructions and published protocols [22,23] with minor modifications. We replaced the splicing vector pSPL3 with pSPL3b provided by Genzyme Corp., Framington, MA, in order to increase exon trapping efficiency as has been reported by Burn et al. [24,60].

Individual BAC clones (500 ng) were completely double digested with *BamHI *and *BglII *and partially digested with *Sau3AI*, respectively, gel-purified and shotgun cloned into the dephosphorylated *BamHI *site of the splicing vector pSPL3b. Recombinant colonies were pooled and plasmid DNA was prepared with a plasmid purification kit (Macherey & Nagel). The DNA was used to transfect COS-7 cells for transient expression of the reporter system by lipofection with lipofectamine plus (Invitrogen). Exon trapping reporter transcripts were examined by extracting total cytoplasmic RNA from COS-7 cells 24 h posttransfection using the RNeasy kit (Qiagen). The RNA was reverse-transcribed with oligo (dT) primer and Superscript II RNase H- reverse transcriptase (Invitrogen) followed by PCR amplification using vector-specific primers (SD6 and SA2 provided by the supplier). To minimize the recovery of products due to cryptic or vector-self splicing, BSTXI digestion of the PCR mixture was performed. Exon trapping libraries were established by subcloning the BSTXI digested PCR products of the transcribed sequences into pAMP10 plasmid vector using the UDG cloning system (Gibco/Life Technologies, now Invitrogen) as recommended by the manufacturer.

Plasmid DNA from recombinant clones was isolated from each exon trapping library and sequenced with vector-cassette-specific primers SD2 and SA4 using ABI-Prism BigDye terminator cycle sequencing kit (Applied Biosystems). Prior to sequencing we included amplification of the inserts of recombinant clones directly from the colonies with primers SD2 and SA4 as a preselection step to minimize sequencing of small exon trapping products. PCR products were analyzed by agarose gel electrophoresis. PCR products exceeding the amplicon size of the empty plasmid vector (>230 bp) were subjected to sequencing. Sequences were compared among themselves and with the vector sequence to identify non-redundant genuine trapped exons.

Primers were designed for unique exon trapped sequences (ETS) exceeding a size of 60 bp (see Additional file 1) and tested for successful PCR amplification on a panel of genomic DNA from their parent BAC clones to verify the genomic origin of the trapped sequences.

Unique ETS were deposited in the Genbank nucleotide database. Accession numbers are given in Additional file 1.

### Analysis and characterization of identified exon trapped sequences

#### Sequence similarity search and in silico mapping on the bovine genome sequence

ETS from BACs specific to BTA6 were used for sequence similarity screening in the currently available 7.15× coverage sequence of the bovine genome at NCBI [61]. The similarity search was performed in the NCBI database [genome (reference only)] using the Basic Local Alignment Search Tool (MegaBLAST) with default parameters. Map positions were retrieved from the current bovine genome sequence assembly map of BTA6 at NCBI (Btau build 4.0 [31]). Generally, each match found in the sequence databases was manually curated. Sequence matches were excluded when revealing less than 95% identity for blast searches.

#### Identification of comparative similarity to human and mouse genomic sequences

In addition to homology search in the bovine genome sequence, ETS were applied to similarity search against human and mouse genomic sequences of the NCBI database [genome (all assemblies)] [62,63] using the cross-species MegaBLAST tool. Comparative sequence similarity screening was performed with low complexity filter and default parameters. Sequence matches were accepted with at least 80% identity across species.

#### Identification of similarity to expressed and coding sequences

Analysis of ETS for protein coding sequences was performed by using the ORF Finder program [64]. Protein and transcript sequence similarity searches were performed at NCBI [65] using BLASTX against the non-redundant protein sequence database (nr) and TBLASTX against the nucleotide collection (nr/nt) and the expressed sequence tags (est) databases with default parameters. Furthermore, transcribed sequences inferred from the ORF Finder analysis were applied to screen the nucleotide collection (nr/nt) and the expressed sequence tags (est) databases using the TBLASTN algorithm. Identity to known bovine sequences was accepted at a threshold of 95% and with more than 70% in the case of non-bovine sequences.

#### Sequence similarity search for non-protein coding RNA

The ETS were analyzed for similarity to known noncoding RNA by screening the comprehensive mammalian noncoding RNA database (RNAdb 2.0, all ncRNA Datasets) using the BLAST algorithm [32].

#### Expression analysis

Expression patterns of identified ETS in cattle were analyzed by RT-PCR on a multi-tissue panel. Total RNA was extracted from nine bovine tissues (liver, small intestine, kidney, thyroid gland, mammary gland, pituitary gland, skeletal muscle, subcutaneous and intestinal fat) isolated from a lactating German Holstein cow. Principally, total RNA was prepared using the Nucleospin II RNA kit (Macherey & Nagel). In case of fat tissues, the RNeasy lipid kit (Qiagen) was applied. Genomic DNA was carefully eliminated by repeated on-column digestion using twice the amount of RNAse-free DNaseI solution the manufacturers recommended in the protocols. The cDNAs were synthesized using the Superscript II RNase H- reverse transcriptase system for first strand cDNA synthesis (Invitrogen,) with oligo (dT) primer according to the manufacturer's instructions.

Quality check-up of the prepared cDNA for contamination with genomic DNA was performed by PCR using primers spanning intron 4 of the bovine PPARGC1A gene. Primers, forward 5'-AAGAAGCTCTTACTGGCACC-3' and reverse 5'-ATGTTGTGTCTGCGATTGTG-3', generated fragments of 318 bp in cDNA and 1177 bp in genomic DNA [66]. Expression of glyceraldehyde-3-phosphate dehydrogenase (GAPDH) as a control gene was analyzed in cDNA of all tissues (forward primer: 5'-TACATGGTCTACATGTTCCAGTATG-3', and reverse primer: 5'-CAGTCTTCTGGGTGGCAGTGATG-3', amplicon: 440 bp). Tissue-specific expression profiling of the identified putative transcripts was performed with primers given in Additional file 1. Bovine genomic DNA was tested to assess amplification on genome level.

## Authors' contributions

RW performed BAC library screening, exon trapping, analyses on genome sequence databases, and expression analyses. TG performed FISH mapping of BAC clones. AE supported database and annotation analyses. RW and CK wrote the manuscript. All authors read and approved the final manuscript.

## Supplementary Material

Additional file 1 **Unique putative exon trapped sequences (ETS) from BAC clones mapped to bovine chromosome 6 (BTA6)**. The data provided represent BTA6 markers used for BAC library screening, BAC clones subjected to exon trapping and details of the isolated ETS (e.g., accession numbers, primers). Locus: region-specific sequence (markers) used for BAC library screening. Click here for file

Additional file 2 **Sequence similarity of identified unique exon trapping sequences (ETS) specific to bovine chromosome 6 (BTA6)**. Genomic positions on BTA6 and insert sizes of BAC clones were determined according to the bovine genome assembly Btau4.0 at NCBI by *in silico *screening of BAC end sequences as described previously [9]. Chromosomal positions of ETS were determined by *in silico *sequence similarity screening to the bovine genome sequence database and chromosome alignment to the bovine genome assembly Btau4.0 at NCBI [31,61]. Comparative sequence similarity of ETS was determined by screening across sequence resources of the human and mouse genomes [62,63]. Similarity to ESTs from *Ovis aries *and *Sus scrofa *and to predicted *Bos taurus *gene models were inferred from the NCBI MapViewer [31] including the additional map options rnaOar, rnaSsc and *ab initio*. Column 5: UN: unassigned. Column 7: Altern. assembly: human genome assembly based on Celera assembly. Click here for file
