# Supplementary material for: Novel transcripts discovered by mining genomic DNA from defined regions of bovine chromosome 6
Source: BMC Genomics. 2009 Apr 24;10:186. doi: 10.1186/1471-2164-10-186 (PMC2681481; doi:10.1186/1471-2164-10-186)
Supplement: Additional file 1 — Unique putative exon trapped sequences (ETS) from BAC clones mapped to bovine chromosome 6 (BTA6). The data provided represent BTA6 markers used for BAC library screening, BAC clones subjected to exon trapping and details of the isolated ETS (e.g., accession numbers, primers). Locus: region-specific sequence (markers) used for BAC library screening. [file 1471-2164-10-186-S1.pdf]

Locus	BAC ID	Clone ID	Insert Size [bp]	ETS_ID	Accession number	Primer (forward)	Primer (reverse)
**FBNS4**	BBI_750F05243	ES4/7	63	20835	ET201800	CAA AAA TGG ACA AAA ACA AAG ATG	GGT TTT TTG ACA ACT TTC GAT G
		ES4/10	120	20837	ET201805	C TTT GCG ACC CCA TGA ACC	TGG ACA TGA GTT TGA GTG AAG
		ES4/35	71	20849	ET201802	CAG CGG GGT TGT TAA TGA AG	TCC CTG TGG AAA GAA CTG C
		ES4/56	114	20853	ET201801	AGT GGG GCT GTC TGT CTC AG	ACT GTG ACT CGG GGG ATG G
		EB4/9	143	20858	ET201803	AGA TGA CAA AGC TAC GCT GG	TG TGA GGG TGG TAC TGT GTC
		EB4/27	213	20866	ET201804	GGT CTT TCT ATT TTG CTT CGG	TGT CTG GGA GCA TCT TCT TTG
**BMS382**	BBI_750F06108	EB7/6	67	25314	ET201811	ATT AGA TAA TCT GTT CCA CTT C	CTC AAG TCA TAA ATG TGG AAC
		EB7/8	108	25315	ET201810	ATC CAG ATG TAG CTG CAT ACG	TCA AAG GGG TAG TTT CCT TGC
		EB7/89	86	25324	ET201812	CAA TGA TAA TTT CGG TCC GTC	GTT CAT GCA GTA CTA TTA GCC
		ES7/17	121	25614	ET201813	GGT CAT CTT ACT AGT GTA TGC	TCC CAG ATG GTT CTA CAA GG
		ES7/30	105	25618	ET201809	GGT GGT ATC ATC TAC ATG CC	CAG GAC ACG TCA TGC AAA G
		ES7/57	121	25624	ET201806	AGA AAT CAG ACA ACT TTA ATC C	TCG CCT TGC AGT TAT TTG TC
		ES7/76	88	25626	ET201807	TTA GTC GGC CAA GTC TAT TC	TCA AGT TCA AAG GCA GTT TTC
		EB9/10	162	25599	ET201819	TCT TTC CAT GAG AGA GAT GAG	TCT TTC CAT GAG AGA GAT GAG
		EB9/58	176	25609	ET201820	GCT GAA AAT GGG TTC TTG TC	CTC CGA GAT TTA ATT TGG CAC
		ES7/12	35	25612	ET201817	No primer	No primer
		ES7/13	46	25613	ET201815	No primer	No primer
		ES7/32	46	25619	ET201814	No primer	No primer
		EB7/11	118	25316	ET201808	No primer	No primer
		EB7/21	53	25319	ET201816	No primer	No primer
		EB7/5	45	25313	ET201818	No primer	No primer
**FBN12**	BBI_750M16219	ES2/2	89	20308	ET201827	CGT AGC CTG GAT AAA TGA AGT TG	TTT GTG TTC CCA GAA GGT AGC
		ES2/3	84	20309	ET201826	CAA TTC CTC GGT TTT GTT TTG	GAA GTC CCT TAA TTG TTG ATT G
		ES2/5	87	20311	ET201825	GAA GAA AAT AAA ATA TAC AGA CTC	GGT GAT ATG GTC CAG GTG TC
		ES2/56	112	20687	ET201821	ACT CTT CTG TCC ATG GGA TTC C	AGG CGA TGC AGG TTT GTT TTC
		ES2/113	79	21395	ET201823	CAT GGA GAT CCA AGA GTA AGC	AGG TTT TAC CCC ACA GAC TTC
		ES2/8b	78	20565	ET201828	ATT TCC ATT TTA ATG ATG AGG AC	CTG TAT GAT TCA GGA CAA AGT G
		ES2/60	197	20691	ET201829	CCC NGT AGA AGG AAA TGG CAC	TCT TAT GAT GGG TGC TGG ATG
		ES2/7b	76	20708	ET201822	CTC AGC TGG TAA GAA TCT GCG	ATC TTC CTG ACC CAG GAA TG
		EB9/8	116	25598	ET201824	CAC ATT CTT TAC CAG CTG AG	AAA ACA TTC TTA GCC TGC TTG
**BM143**	BBI_750F22162	EB3/2	69	20395	ET201835	CAG CTT AGT GCT TGA GGT CC	AGT TCT CTT TTG CAT GTA GGG
		EB3/5	120	20396	ET201833	AGT GAG TCA AGA AAG CCA GTG	CAT AAT GAA GTT CTG CTC TCC
		EB3/12	45	20400	ET201834	No primer	No primer
		ES3/3	95	21685	ET201832	CAT TAC TGT GTC TGA AGT CTC	TCT TCA AGG AAA GAA TTC AAC

		ES3/9	185	21687	ET201837	CAA ACT CAA GAA CAA TGC TAT G	TGG GTT TGA TTC CTG GGT TG
		ES3/10	176	21688	ET201836	AGA GAT GGG GGT TCA GTC C	TGA TGT TGT TGA AGT TTA GTT G
		ES3/35	315	21696	ET201831	CAG TTA TAT ACA GTA CCC TGG	ATG TAG CAT TTC CTC CTC TTG
		ES3/38	67	21697	ET201830	GCA TGA AAA ATC AAA CCA AAC AT	ATT CTT ATA GCA GGT TCC CAG
**FBNS10**	BBI_750J07162	ES6/11	130	25283	ET201842	AAG AAA CTC TTC ATC ATC CAG	TGA GCA TGA ATG TGG GTT TC
		ES6/22	116	25285	ET201839	GTT AGG AGC CTT CTG GTG TG	AGA AGA ATG TAG ACA GGT AGG
		ES6/72	92	25292	ET201841	GTG CCT GTC TCC TTC TTT TG	CAC AGT CAT GTG AAT TCA TG
		ES6/78	117	25294	ET201843	GGC ATG ATG AGG GAT CCT G	TCC ATC CTG ACT TGT TTT CTC
		ES6/36	47	25257	ET201838	No primer	No primer
		EB6/6	94	25299	ET201840	AAA TAA AAC ATG TAA TAC CAA AG	CTG TCT CCT ATA ATT GGA TTG
		EB6/25	239	25303	AY839826	GAA GGC CAA AAT ATC ACC TG	TCT TGT TAT CTG TAC CGC TTG
		EB6/78	256	25310	AY839825	CGA CAC TCA GAG TCA TGT G	CTC TTT TGG GAT TTT GAA CAG
**BL1099**	BBI_750C04346	EB10/6	112	22258	ET201844	GTA AGC TGA GTC CCC AGT G	ACT TTG GTC CTA TCA CGA ATC
		ES10/10	149	26267	ET201847	AAC TGA TGA TTA TAC AAC TCA G	ACA CTC CCA TAA TAT ACA TTC C
		ES10/28	191	23268	ET201840	ACG AAT GGA CCT CAT AGA AGC G	TGA ACG TGG AGT AGC TCC TCT C
		ES10/73	160	26271	ET201845	GAA CTG CAT ACA GCA TAG TCC	GTT GGC AAA GTG ATG TCT CTG
**TGLA37**	BBI_750B13346	EB15/5	84	22268	ET201850	C ACT TGA CCA TCT GGA AGT TTA	CTC ACA CAC TAG TAA AGT AAT G
		EB15/6	97	22269	ET201851	AGA CTC TGA TGC TGG GAG GA	GAG TCA GTG ATG CCA TCC AG
		EB15/87	98	22277	ET201852	GCA GGA AAG AGC AGA TTG TC	CAC ATC AGG GCA GGG GCA G
		ES15/54	176	26264	ET201848	ATC ATT GAT CTG GAA AGA TGC	CAC ACA GGC TTC AGT GAT TG
		ES15/24	206	26263	ET201849	CAC AGG CTC TCC ACC CTC C	GGT ACA AAA GCT GCA TCA TAG
**TGLA37**	BBI_750E07177	EE07177-1	95	15335	ET201854	ACA AAA TCT TAG TCG ATG TTG TC	TTT ATG TAT GAC CAC CAG CAA C
		EE07177-5	218	15338	ET201855	CAA CAA CAC AAG AGA GAT GGT C	CCG ATC TTG TTT TTG CTG GC
		E207177/2-2	350	16062	ET201853	AAA TTT GGC TTT GGA ATA TGG	TTG GCT GCA AAG AAT AAT CAA
		E207177/2-6	55	16065	ET201856	No primer	No primer
**BMS518**	BBI_750E20138	EB20/1	137	26308	ET201857	GCA ATA TAT ATT AGC TCC TGG	GTC CAT CAA GTC AAT GAT GCC
		EB20/8	90	26342	ET201858	AAT GGA TTT CTT GCA GCA CC	GTA CAG TCT TCC TTG TCT CC
		EB9/1	225	25582	ET201860	GGT TGA CCA GTT TTC CCA G	ACA ACT TAT CTT CAA CAA AGG
		ES9/3	59	25584	ET201859	CAT CCA TTA GAT GCC TCC TC	TCT TGA AAA GTA CAG TAG TTC
		EB20/45	42	26352	ET201861	No primer	No primer
**FBNS13**	BBI_750K15348	EB18/6	89	25328	ET201862	AAT CCT TTG CAG AAA AAC TTT G	ATA GTT TCT CTC CAT AGA TTA C
		EB18/8	94	25329	ET201864	ATG TGT GGC ATC ATG AAT CTC	TAT CTG TTG TGA CCA TTT TCC
		EB18/49	109	25336	ET201863	TTT GAA AGT GCT CAA CTG GAG	ACA TCC TGG AAT GTG AAG TCC
**FBN13**	BBI_750M0258	EM0258-57	162	14638	ET201867	CTT TGAGCC AAG AGT AAC TGC	TCT TTT GTC CAT GGG ATT TTC
		E2M0258-4	68	15495	ET201866	CTT CCC TGA TGG CTC AGA TGA	AAT AGA ATC CAG GTC TCT CAC AG

		E2M0258-6	126	15498	ET201865	ACA GTC AGC AAA AAC AAG ACT G	GAA TGG TCT AGT GGT TTT CCT T
**FBN13**	BBI_750B0172	E2B0172-2	142	15239	ET201868	CAA TTC TCT GTG CCA AAC TG	GAG ACA TAA GTG CCA TTG GTA G
		E2B0172/1-12	69	16035	ET201869	TTT ATT TAA GCT GGA AGA GAC	GTC TGA ATT TTA TGT TGG GG
		E3B0172/1-14	241	16037	ET201870	TTA TGG CAA ATA GAT GGA AAA C	TTT TCT TAA AAG AAG CAA GCC
		E3B0172/1-18	143	16041	ET201871	TC TGT CCA TGG AAA TCT G	GTC TAG TGG CTC AGA TGG C
		E3B0172/1-20	233	16042	ET201872	CAG TCG TGT CTG ACC CCA C	TGC CCA TAG GGT CAC ACA G
		E2B0172-9	57	15246	ET201873	GCC TTA GTG AAG AAC TAC	GGT CTG GTA TCT CTT ATG TCT
		E3B0172/1-35	172	16667	ET201874	TGC ATC TGC AGT AGA TAA ACC	TTT ACA CTG ACC CTG TAG ATC C
**BM4528**	BBI_750A1391	ES5/5	203	20746	ET201881	GCT CAC CTG TCC TTC ACT ATC	TTC GAG ATT TTC CAA ACT CAG
		ES5/12	120	20748	ET201875	TGA TAT TTG TAT ACC TCC AGA C	TTA TAT CAG AAT GCT CTC CAA G
		ES5/13	202	20749	ET201877	CCT GCG CGG AAC CTT ACC	GA AGA GAC TCG AGC GCA AG
		ES5/15	126	20751	ET201882	GAG AGA CAC AGG AAG GAG AAT G	TGA CTG TTG GTC TTT AGC ACT G
		ES5/23	218	20758	ET201883	GGA GGT TTG TTC TAC TGG AGG	CTG TGG CCA CTG CTG AGT TTC
		EB5/9	106	23434	ET201878	GAC TCT GAT GCT GGG AGG AAT	CCA TCG AGT CAG TGA TGC C
		EB5/12	162	23435	ET201879	GAT TCT GCA GGC AAG GCT G	CTG ATG GTA AAG AAT CTG CTG
		EB5/66	94	23447	ET201880	CTC CTG AAT CAG AAC AGT GAT G	AGG AGG ATG CCT TGG GAG
		EB5/16	74	23437	ET201884	No primer	No primer
		EB5/18	54	23438	ET201876	No primer	No primer
**FBN14**	BBI_750C1821	EC1821-6	212	15328	ET201889	GAA GGA CTG ATG AGA AAC AAT G	TTC TTC TCA TCT TTC ATC CAT C
		EC1821/2-2	108	16048	ET201888	CCA ACA AGG GTA GAA GAA AAG	ATG CAG GTT CGA TCT CCG TG
		EC1821/2-4	224	16050	ET201887	CAG TTT GGA TCC CTG AGT CTG	TCA ACT CTT TGC AAG CCA CAG
		EC1821/2-8	48	16053	ET201885	CAG CAG ACG CTT TAC CCT C	CTC CAT ACA CAG CGG CTT C
		EC1821/1-30	151	16662	ET201886	AAA TTT GGC TTT GGA ATA TGG	TTG GCT GCA AAG AAT AAT CAA
