# Supplementary material for: Novel transcripts discovered by mining genomic DNA from defined regions of bovine chromosome 6
Source: BMC Genomics. 2009 Apr 24;10:186. doi: 10.1186/1471-2164-10-186 (PMC2681481; doi:10.1186/1471-2164-10-186)
Supplement: Additional file 2 — Sequence similarity of identified unique exon trapping sequences (ETS) specific to bovine chromosome 6 (BTA6). Genomic positions on BTA6 and insert sizes of BAC clones were determined according to the bovine genome assembly Btau4.0 at NCBI by in silico screening of BAC end sequences as described previously [9]. Chromosomal positions of ETS were determined by in silico sequence similarity screening to the bovine genome sequence database and chromosome alignment to the bovine genome assembly Btau4.0 at NCBI [31,61]. Comparative sequence similarity of ETS was determined by screening across sequence resources of the human and mouse genomes [62,63]. Similarity to ESTs from Ovis aries and Sus scrofa and to predicted Bos taurus gene models were inferred from the NCBI MapViewer [31] including the additional map options rnaOar, rnaSsc and ab initio. Column 5: UN: unassigned. Column 7: Altern. assembly: human genome assembly based on Celera assembly. [file 1471-2164-10-186-S2.pdf]

Locus on BTA6	BAC ID with insert size and BAC position on BTA6 scaffold [Mb]	ETS_ID	Similarity to bovine genome sequence	Position [Mb]	Similarity to other transcripts	Similarity to other genome sequences
**FBNS4**	BBI_750F05243 (99 Kb) NW_001495159.2: 41.414-41.513	20835	NW_001495159.2, KCNIP4 (NM_001076935, exon 5),	BTA6: 41.423	Exon 13 of human KCNIP4	
		20849	NW_001495159.2, KCNIP4, exon 2	BTA6: 41.440	Exon 9 of human KCNIP4	
		20853	NW_001495159.2, KCNIP4, intron 1	BTA6: 41.464		
		20858	NW_001495159.2, KCNIP4, intron 1	BTA6: 41.495		Intron 4 of human KCNIP4
		20837	NW_001495159.2, KCNIP4, intron 1	BTA6: 41.508		Human genomic contigs, e.g. NW_921665, altern. assembly
		20866	-----------------------------	UN	11 bovine ESTs (e.g. DY055462), exons 11 and 12 of human KCNIP4	
**BMS382**	BBI_750F06108 (110 Kb) NW_001495159.2: 42.091-42.201	25626	NW_001495159.2, 584.6 Kb 5' to KCNIP4	BTA6: 42.107	Ovine EST EE834255, Bovine predicted transcript hmn 107334 (gnomon model)	
		25624	NW_001495159.2, 586.2 Kb 5' to KCNIP4	BTA6: 42.109	Ovine EST EE834255, Bovine predicted transcript hmm 107334 (gnomon model)	
		25316	NW_001495159.2, 591 Kb 5' to KCNIP4	BTA6: 42.114	Ovine EST EE834255	Human genomic contigs, e.g. NW_923238, altern. assembly
		25618	NW_001495159.2, 592.9 Kb 5' to KCNIP4	BTA6: 42.116	Ovine EST EE834255	
		25599	NW_001495159.2, 594.7 Kb 5' to KCNIP4	BTA6: 42.119	Ovine EST EE834255	
		25609	NW_001495159.2, 611.4 Kb 5' to KCNIP4	BTA6: 42.134	Bovine ESTs DY149610, DY194833, DY1944707 assigned to NW_00149515 Ovine EST EE834255	
		25315	NW_001495159.2, 622.5 Kb 5' to KCNIP4	BTA6: 42.145	Ovine EST EE834255	
		25319	NW_001495159.2, 649.2 Kb 5' to KCNIP4	BTA6: 42.172	Ovine ESTs EE834255 and EE870115	Human genomic contigs on multiple chromosomes
		25314	NW_001495159.2, 652.9 Kb 5' to KCNIP4	BTA6: 42.176	Ovine EST EE834255	
		25324	NW_001495159.2, 655.2 Kb 5' to KCNIP4	BTA6: 42.178	Ovine EST EE834255	
		25614	NW_001495159.2, 658.5 Kb 5' to KCNIP4	BTA6: 42.182		
		25619	NW_001495159.2, 662.3 Kb 5' to KCNIP4	BTA6: 42.186		
		25313	-----------------------------	UN		
		25612	-----------------------------	UN		
		25613	-----------------------------	UN		
**FBN12**	BBI_750M16218 (64 Kb) NW_001495159.2: 43.3825-43.4460	20687	NW_001495159.2, 29.8 Kb 5' to GPR125, (XM_614752.3)	BTA6: 43.383		Human genomic contigs, e.g. NW_921594, NW_922185, altern. assembly
		20708	NW_001495159.2, 44.1 Kb 5' to GPR125, 44.4 Kb 3' to GBA3 (NM_001787544.3)	BTA6: 43.397		Human genomic contigs, e.g. NW_922099, altern. assembly
		21395	NW_001495159.2, 63 Kb 5' to GPR125, 25.5 Kb 3' to GBA3	BTA6: 43.416	Bovine predicted transcript hmm 133334 (gnomon model)	
		25598	NW_001495159.2, 70.4 (78.3) Kb 5' to GPR125, 18.1 (10.2) Kb 3' to predicted	BTA6: 43.423 and 43.431	Bovine predicted transcript hmm 133334 (gnomon model)	Human genomic contig, NW_921914, altern. Assembly

			GBA3 (XM_)			Mouse genomic contig NT_39305 on MMU5, 107 Kb 5’ to Gpr125, 1.29 Mb 3’ to pargc1a
		20309	NW_001495159.2, 76 Kb 5’ to GPR125, 12.5 Kb 3’ to GBA3	BTA6: 43.429	Bovine predicted transcript hmm 133334 (gnomon model)	
		20308	NW_001495159.2, 77 Kb 5’ to GPR125, 11.5 Kb 3’ to GBA3	BTA6: 43.4301	Bovine predicted transcript hmm 133334 (gnomon model)	
		20311	NW_001495159.2, 77.1 Kb 5’ to GPR125, 11.4 Kb 3’ to GBA3	BTA6: 43.4302	Bovine predicted transcript hmm 133334 (gnomon model)	Human genomic contigs, e.g. NW_921594, altern. assembly
		20565	NW_001495159.2, 79 Kb 5’ to GPR125, 9.4 Kb 3’ to GBA3	BTA6: 43.432		Human genomic contigs on HSA18 and HSA12
		20691	UN, partiell repetitive	UN		
**BM143**	BBI_750F22162 (76 Kb) NW_001495167.2: 44.107-44.183	21697	NW_001495167.2	BTA6: 44.114		
		21696	NW_001495167.2	BTA6: 44.1165 and 44.118		Human genomic contig NT_006316 on HSA4, 442 Kb 5’ to LOC643751 , 625 Kb 3’ to PPARGC1A, Mouse genomic contig NT_39305 on MMU5, 733 Kb 5’ to Gpr125, 663 Kb 3’ to Ppargc1a
		21688	NW_001495167.2	BTA6: 44.1178		Human genomic contigs, e.g. NW_922185, altern. assembly
		20396	NW_001495167.2	BTA6: 44.134		
		21685	NW_001495167.2	BTA6: 44.141		
		20400	NW_001495167.2	BTA6: 44.168		
		20395	NW_001495167.2	BTA6: 44.170		
		21687	NW_001495167.2	BTA6: 44.175		Human genomic contigs, e.g. NW_922900, altern. assembly
**FBNS10**	BBI_750J07162 (66 Kb) NW_001495167.2: 45.374-45.440	25285	NW_001495167.2, 554.4 Kb 5’ to PPARGC1A (NM_177945.3)	BTA6: 45.393		
		25657	NW_001495167.2, 77.9 Kb 3’ to DHX15 (NM_001098018.3)	BTA6: 45.407		
		25310	NW_001495167.2, 73.6 Kb 3’ to DHX15	BTA6: 45.411		Human genomic contig NT_006316 on HSA4, 544 Kb 5’ to PPARGC1A, 37 Kb 3’ to LOC729175
		25303	NW_001495167.2, 580.3 (581.1, 581.5) Kb 5’ to PPARGC1A, 65.8 (65.1, 64.7) Kb 3’ to DHX15	BTA6: 45.4191, 45.4198, and 45.4202		Human genomic contig NT_006316 on HSA4, 552 Kb 5’ to PPARGC1A, 29 Kb 3’ to LOC729175
		25299	NW_001495167.2, 59.8 Kb 3’ to DHX15	BTA6: 45.425		Human genomic contig NT_006316 on HSA4, 560 Kb 5’ to PPARGC1A, 22 Kb 3’ to LOC729175 Mouse genomic contig NT_39305 on MMU5, 240 Kb 5’ to Ppargc1a, 56 Kb 3’ to Dhx15
		25292	NW_001495167.2, 54.6 Kb 3’ to DHX15	BTA6: 45.430		

		25283	NW_001495167.2, 49.3 Kb 3’ to DHX15	BTA6: 45.435		
		25294	NW_001495167.2, 47.8 Kb 3’ to DHX15	BTA6: 45.437	Bovine EST DY067729<br>Bovine predicted transcript hmm 183334 (gnomon model)	
**BL1099**	BBI_750C04346 (60 Kb)<br>NW_001495169.2: 49.074-49.134	26271	NW_001495169.2	BTA6: 49.080	Bovine predicted transcript hmm 281334 (gnomon model)	Human genomic contig NW_922643, altern. assembly
		22258	NW_001495169.2	BTA6: 49.088		
		26268	NW_001495169.2	BTA6: 49.118		
		26267	NW_001495169.2	BTA6: 49.123		
**FBNS13**	BBI_750K15348 (865 Kb)<br>NW_001495170.2: 50.439-51.304	25328	NW_001495170.2, 228.8 Kb 3’ to PCDH7 (XM_864701)	BTA6: 51.287		
		25329	NW_001495170.2, 274.5 Kb 3’ to PCDH7	BTA6: 51.241		
		25336	NW_001495170.2, 268.5 Kb 3’ to PCDH7	BTA6: 51.247		
**BMS518**	BBI_750E20138 (50 Kb)<br>NW_001495170.2: 51.287-51.337	25582	NW_001495170.2, 208.3 (209.0) Kb 3’ to PCDH7	BTA6: 51.3069 and 51.3075		
		25584	NW_001495170.2, 202.4 Kb 3’ to PCDH7,	BTA6: 51.313		
		26352	NW_001495170.2, 191.1 Kb 3’ to PCDH7	BTA6: 51.3248		
		26342	NW_001495170.2, 191 Kb 3’ to PCDH7	BTA6: 51.325		
		26308	NW_001495170.2, 184.4 Kb 3’ to PCDH7	BTA6: 51.332	Bovine ESTs assigned to a number of chromosomes	Human genomic contigs, e.g. NW_921858, altern. assembly
**TGLA37**	BBI_750E07177 (77 Kb)<br>NW_001495170.2: 52.453-52.530	16065	NW_001495170.2, 467.3 Kb 5’ to PCDH7	BTA6: 52.467		
		15338	NW_001495170.2, 514.9 Kb 5’ to PCDH7	BTA6: 52.5145		Human genomic contig NW_921594, altern. assembly
		15335	NW_001495170.2, 516.9 Kb 5’ to PCDH7	BTA6: 52.516		
		16062	NW_001495170.2, 523.2 Kb 5’ to PCDH7	BTA6: 52.523		Human genomic contig NW_921594, altern. assembly
**TGLA37**	BBI_750B13346 (72 Kb)<br>NW_001495170.2: 52.458-52.530	26263	NW_001495170.2, 469.4 (468.9) Kb 5’ to PCDH7	BTA6: 52.4687 and 52.4683)		
		26264	NW_001495170.2, 524.5 Kb 5’ to PCDH7	BTA6: 52.524		
		22268	NW_001495170.2, 1,013.6 Kb 5’ to PCDH7	BTA6: 53.013		Human genomic contig NW_921789, altern. assembly
		22269	NW_001495170.2, 1,060.3 Kb 5’ to PCDH7	BTA6: 53.059		Human genomic contigs, e.g. NW_921858, NW_921665, altern. assembly
		22277	NW_001503872.2	UN	Bovine predicted transcript hmm 2103723 (gnomon model)	Mouse genomic contig NT_39606 on MMU14, Polr3d
**FBN13**	BBI_750M0258 (198 Kb)<br>NW_001495175.2: 56.090-56.288	15498	NW_001495175.2, partial repetitive sequence	BTA6: 56.100		Human genomic contig NW_921594, altern. assembly
		15495	NW_001495175.2 and NW_001495179.2	BTA6: 56.115 and 56.253		Human genomic contig NW_921914, altern. assembly
		14638	NW_001495179.2	BTA6: 56.278		
**FBN13**	BBI_750B0172 (92/83 or 48/222Kb)<br>NW_001495175.2/NW_001495179.2: 56.128/56.267-56.175/56.350	16042	NW_001495179.2	BTA6: 56.280		Human genomic contig NW_921699, altern. assembly

		16037	NW_00149517.2	BTA6: 56.2878 and 56.2883		Human genomic contig NW_922974, altern. assembly
		15239	NW_001495179.2	BTA6: 56.289		
		15246	NW_001495179.2	BTA6:56.292	Bovine ESTs DN840825, DN845774	
		16035	NW_001495179.2	BTA6: 56.302	Porcine EST EW1959132	
		16667	NW_001495179.2	BTA6: 56.304	Bovine predicted transcript hmm 37344 (gnomon model) and hypothetical locus LOC100140872	Human genomic contigs on HSA10 and HSA8, PHYHIPL Mouse genomic contigs on MMU10 and MMU14, Phyhipl
		16041	--------------------------	UN		Human genomic contigs NW_921665, NW_921517, altern. assembly
**BM4528**	BBI_750A1391 (33 Kb) NW_001495186.2: 65.832-65.865	20748	NW_001495186.2, 8.0 Kb 5’ to KCTD8, (XM_590048)	BTA6: 65.588	Bovine predicted transcript hmm 325344 (gnomon model)	
		20751	NW_001495186.2, KCTD8, intron	BTA6: 65.835		Human genomic contigs on HSA6, HSA12, HSA5
		20746	NW_001495186.2, KCTD8, intron	BTA6: 65.853		Human genomic contig, NW_922610, altern. assembly, pred Hmm438037
		23447	NW_001495186.2, KCTD8, intron	BTA6: 65.8545		
		23435	NW_001495186.2, KCTD8, intron	BTA6: 65.860		Human genomic contigs, e.g. NW_922226, altern. assembly
		20758	NW_001495186.2, KCTD8, intron	BTA6: 65.865		Human genomic contigs, e.g. NW_921858,, NW_921665 altern. assembly
		20749	NW_001495186.2, KCTD8, intron	BTA6: 65.879		
		23438	NW_001495186.2, 2.0 Kb 5’ to KCTD8	BTA6: 65.882	Bovine predicted transcript hmm 325344 (gnomon model)	
		23437	NW_001503872.1	UN	Bovine predicted transcript hmm 2102733 (gnomon model) and hypothetical locus LOC788223	Mouse genomic contig NT_39606 on MMU14, Pol3rd
		23434	NW_001502282, NW_001501795, NW_001495173.1	UN BTA6:46.99	Repetitive motifs in bovine ESTs assigned to multiple chromosomes	Human genomic contigs, e.g. NW_921858,, NW_921665 altern. assembly
**FBN14**	BBI_750C1821 (83 Kb) NW_001495201.2: 78.415-78.498	16053	NW_001495201.2	BTA6: 78.430		
		16662	NW_001495201.2	BTA6: 78.435		Human genomic contigs, e.g. NW_921665 and Mouse genomic contig NW_001037873, altern. assemblies
		16048	NW_001495201.2	BTA6: 78.448		Human genomic contigs, e.g. NW_921665 altern. assembly
		15328	NW_001495201.2	BTA6: 78.4574/78.4576 and 78.4612/78.4614		Human genomic contig NT_022778 on HSA4, 144 Kb 5’ to LOC644419, 1.2 Mb 3’ to LOC644484
		16050	NW_001495201.2	BTA6: 78.471		Human genomic contigs, e.g. NW_922185, altern. assembly
